# A wild derived quantitative trait locus on mouse chromosome 2 prevents obesity

**DOI:** 10.1186/1471-2156-11-84

**Published:** 2010-09-23

**Authors:** Md Bazlur R Mollah, Akira Ishikawa

**Affiliations:** 1 Laboratory of Animal Genetics, Division of Applied Genetics and Physiology, Department of Applied Molecular Bioscience, Graduate School of Bioagricultural Sciences, Nagoya University, Aichi 464-8601, Japan

## Abstract

**Background:**

The genetic architecture of multifactorial traits such as obesity has been poorly understood. Quantitative trait locus (QTL) analysis is widely used to localize loci affecting multifactorial traits on chromosomal regions. However, large confidence intervals and small phenotypic effects of identified QTLs and closely linked loci are impeding the identification of causative genes that underlie the QTLs. Here we developed five subcongenic mouse strains with overlapping and non-overlapping wild-derived genomic regions from an F2 intercross of a previously developed congenic strain, B6.Cg-*Pbwg1*, and its genetic background strain, C57BL/6J (B6). The subcongenic strains developed were phenotyped on low-fat standard chow and a high-fat diet to fine-map a previously identified obesity QTL. Microarray analysis was performed with Affymetrix GeneChips to search for candidate genes of the QTL.

**Results:**

The obesity QTL was physically mapped to an 8.8-Mb region of mouse chromosome 2. The wild-derived allele significantly decreased white fat pad weight, body weight and serum levels of glucose and triglyceride. It was also resistant to the high-fat diet. Among 29 genes residing within the 8.8-Mb region, *Gpd2, Upp2, Acvr1c, March7 *and *Rbms1 *showed great differential expression in livers and/or gonadal fat pads between B6.Cg-*Pbwg1 *and B6 mice.

**Conclusions:**

The wild-derived QTL allele prevented obesity in both mice fed a low-fat standard diet and mice fed a high-fat diet. This finding will pave the way for identification of causative genes for obesity. A further understanding of this unique QTL effect at genetic and molecular levels may lead to the discovery of new biological and pathologic pathways associated with obesity.

## Background

Obesity is a multifactorial disease and is influenced by genetic and environmental components and their interactions. It is an important predisposing factor of serious chronic diseases including type 2 diabetes, hypertension, cardiovascular disease, and some forms of cancer [1]. Despite the identification of several Mendelian genes related to obesity, our understanding of the genetic architecture of the common form of obesity is poor. In recent years, quantitative trait locus (QTL) analysis has become a standard procedure for localizing loci affecting such multifactorial disease traits on chromosomal regions. QTL mapping has revealed many loci related to body weight, growth, obesity and diabetes as reviewed elsewhere [2], and the number of identified loci is increasing day by day. However, identification of causative genes contributing to variation in traits has proven extremely difficult for three main reasons. First, the confidence intervals of identified QTLs remain wide, usually spanning 10-50 cM and possibly harboring hundreds or thousands of genes [3,4]. Second, each locus often explains only a fraction of the phenotypic variation [5]. Third, a single QTL is likely to be composed of multiple linked QTLs [6,7]. Among the approximately 2000 QTLs reported in rodents, only a few percentages have been cloned, indicating the necessity to confirm the presence of the identified QTL by initial genome-wide analysis and to reduce the confidence interval of the QTL to a level allowing positional cloning by fine mapping [3].

Among several approaches proposed to fine-map a QTL, the use of congenic and subsequently developed subcongenic strains is a traditional but powerful method because it allows multiple tests for phenotypic effects on a uniform genetic background except for the QTL region [7-12]. Recently, a new approach with integration of linkage analysis and gene expression profiling has been proposed to search for and identify candidate genes underlying QTLs [11,13-16]. In this study, we thus combined the conventional congenic-subcongenic fine-mapping approach and microarray analysis.

In a previous study, we developed a congenic strain, B6.Cg-*Pbwg1*, with a 44.1-Mb genomic region derived from the Philippine wild mouse, *Mus musculus castaneus*, onto the genetic background of a common inbred strain, C57BL/6J (B6), and subsequent QTL analysis in an F2 intercross between B6.Cg-*Pbwg1 *and B6 revealed several closely linked QTLs affecting body weight gain, lean body weight and obesity within the 44.1-Mb region harboring *Pbwg1*, a growth QTL on mouse chromosome 2 [6,17]. Four obesity QTLs (*Pbwg1.5*, *Pbwg1*.6, *Pbwg1.7 *and *Pbwg1.8*) were mapped to a small interval from 39.0 to 75.6 Mb on chromosome 2 [6]. In this study, we developed a series of subcongenic strains derived from B6.Cg-*Pbwg1 *to fine-map the obesity QTLs and evaluated their effects on standard and high-fat diets. We also searched for candidate genes of the QTLs by microarray analysis.

## Results

### Development of subcongenic strains

**Figure 1:**
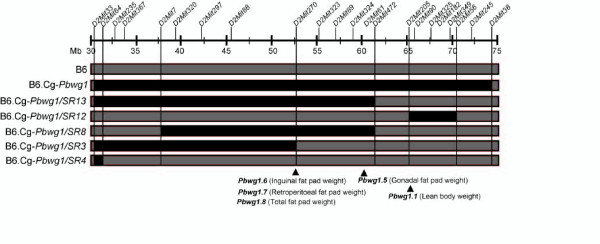
**Relative genomic intervals of subcongenic strains developed from B6.Cg-*Pbwg1*.** The black bar indicates the minimum genomic region derived from the *Mus musculus castaneus *mouse, whereas the gray bar indicates a region from the C57BL/6J (B6) mouse. Distal and proximal boarders of each subcongenic strain are arbitrarily drawn by vertical lines. The physical map position (Mb) of microsatellite markers is shown on the horizontal line (Table 1). The triangle indicates the position of the peak LOD score for previously identified QTLs affecting fat pad weights and lean body weight [6].

F2 mice obtained from an intercross of B6 and B6.Cg-*Pbwg1 *strains were screened with 25 microsatellite markers (Table 1) to detect recombinant mice. The recombinants were mated to fix subcongenic regions. Finally, five subcongenic strains carrying the following wild-derived genomic regions on chromosome 2 were obtained: 30.45-61.54 Mb for B6.Cg-*Pbwg1*/SR13, 65.15-70.48 for B6.Cg-*Pbwg1*/SR12, 38.09-61.54 for B6.Cg-*Pbwg1*/SR8, 30.45-52.76 for B6.Cg-*Pbwg1*/SR3 and 30.45-31.20 for B6.Cg-*Pbwg1*/SR4 (Figure 1).

**Table 1:** Microsatellite markers used for genotyping and refining subcongenic borders

Marker name	Physical map position (bp) ^a^	Genetic map position (cM) ^b^
*D2Mit33*	30457829 - 30458008	17.00
*D2Mit64*	31200277 - 31200449	18.00
*D2Mit235*	32753980 - 32754099	22.50
*D2Mit367*	33488530 - 33488680	26.20
*D2Mit7*	38095233 - 38095379	28.00
*D2Mit320*	39015155 - 39015274	27.30
*D2Mit297*	42461006 - 42461146	29.00
*D2Mit88*	45313143 - 45313324	30.00
*D2Mit270*	52766346 - 52766428	30.50
*D2Mit323*	55163327 - 55163451	31.70
*D2Mit89*	56480405 - 56480598	32.00
*D2Mit433*	57149522 - 57149694	31.70
*D2Mit324*	58783118 - 58783242	32.80
*D2Mit123*	59242475 - 59242615	33.00
*D2Mit61*	60528325 - 60528469	34.00
*D2Mit472*	61544415 -61744509	38.30
*D2Mit205*	65140125 - 65140208	37.00
*D2Mit90*	65403934 -65604017	37.00
*D2Mit325*	67599653 - 67599827	38.30
*D2Mit182*	68710989 - 68711138	38.30
*D2Mit349*	69737962 - 69738092	40.40
*D2Mit327*	69303093 - 69303218	40.40
*D2Mit56*	70488173 - 70688291	41.00
*D2Mit245*	72462129 - 72462229	43.00
*D2Mit38*	74535365 - 74535555	45.00

^a^UCSC Genome Browser (Mouse, July 2007 assembly). ^b^Mouse Genome Database (September, 2009, release 4.3).

### Phenotypic characterization of subcongenics

Since body weight and obesity-related traits are greatly influenced by nutritional environments, these traits were compared among five subcongenic strains and their parental strains, B6.Cg-*Pbwg1 *and B6, fed the same standard chow. In addition, they were compared among a subset of male mice of three selected subcongenic strains and the parental strains fed a high-fat diet.

We performed re-phenotyping using a new cohort of B6.Cg-*Pbwg1 *and B6 mice, different from that in our previous study [6]. The original B6.Cg-*Pbwg1 *congenic strain fed a standard chow diet had a strong phenotypic effect on individual fat pad and total fat pad weights at 13 weeks of age (Table 2), confirming the presence of obesity QTLs identified in our previous study [6].

**Table 2:** Obesity traits in five subcongenics and their parental strains on low-fat standard chow diet

Sex	Strain	n	Body weight (g)	Lean body weight (g)	Inguinal fat pad weight (g)	Gonadal fat pad weight (g)	Retroperitoneal fat pad weight (g)	Total fat pad weight (g)	Adiposity index
Male	B6	45	27.28 ± 0.051	26.23 ± 0.048	0.455 ± 0.005	0.424 ± 0.003	0.164 ± 0.003	1.043 ± 0.009	0.038 ± 0.0003
	B6.Cg-*Pbwg1*	40	26.83 ± 0.063	26.04 ± 0.061	0.336 ± 0.003***	0.338 ± 0.003***	0.116 ± 0.002***	0.790 ± 0.004***	0.031 ± 0.0007***
	B6.Cg-*Pbwg1*/SR13	15	27.69 ± 0.057	26.90 ± 0.056	0.345 ± 0.006**	0.308 ± 0.006***	0.132 ± 0.004	0.785 ± 0.014***	0.035 ± 0.0008***
	B6.Cg-*Pbwg1*/SR12	30	29.00 ± 0.115**	28.07 ± 0.108**	0.393 ± 0.007*	0.390 ± 0.007	0.149 ± 0.003	0.933 ± 0.014*	0.028 ± 0.0008***
	B6.Cg-*Pbwg1*/SR8	39	26.13 ± 0.055*	25.49 ± 0.054*	0.293 ± 0.004***	0.265 ± 0.003***	0.082 ± 0.002***	0.641 ± 0.007***	0.030 ± 0.0014***
	B6.Cg-*Pbwg1*/SR3	16	27.22 ± 0.039	26.19 ± 0.036	0.439 ± 0.003	0.453 ± 0.003	0.176 ± 0.004	1.029 ± 0.009	0.031 ± 0.0001
	B6.Cg-*Pbwg1*/SR4	23	28.01 ± 0.172	26.89 ± 0.157	0.466 ± 0.011	0.470 ± 0.006	0.191 ± 0.007	1.128 ± 0.022	0.034 ± 0.0011

Female	B6	39	21.96 ± 0.044	21.17 ± 0.043	0.359 ± 0.002	0.296 ± 0.002	0.136 ± 0.001	0.791 ± 0.003	0.036 ± 0.0002
	B6.Cg-*Pbwg1*	31	21.24 ± 0.056**	20.63 ± 0.052**	0.266 ± 0.002***	0.234 ± 0.002***	0.108 ± 0.001***	0.608 ± 0.004***	0.029 ± 0.0002***
	B6.Cg-*Pbwg1*/SR13	14	21.03 ± 0.055	20.48 ± 0.053	0.268 ± 0.001***	0.175 ± 0.003***	0.104 ± 0.001*	0.547 ± 0.005***	0.026 ± 0.0003***
	B6.Cg-*Pbwg1*/SR12	30	22.33 ± 0.066	21.55 ± 0.061	0.366 ± 0.003	0.278 ± 0.004	0.130 ± 0.002	0.774 ± 0.005	0.034 ± 0.0002
	B6.Cg-*Pbwg1*/SR8	31	20.02 ± 0.050***	19.59 ± 0.049***	0.233 ± 0.003***	0.129 ± 0.002***	0.063 ± 0.001***	0.425 ± 0.004***	0.021 ± 0.0002***
	B6.Cg-*Pbwg1*/SR3	14	21.81 ± 0.066	21.12 ± 0.067	0.344 ± 0.002	0.289 ± 0.002	0.147 ± 0.001	0.780 ± 0.005	0.036 ± 0.0003
	B6.Cg-*Pbwg1*/SR4	27	22.14 ± 0.046	21.36 ± 0.041	0.375 ± 0.005	0.288 ± 0.005	0.112 ± 0.002*	0.776 ± 0.006	0.035 ± 0.0002

Data are least-squared means ± SE at 13 weeks of age. n, number of mice. Sex separated data were analyzed with a linear mixed model (see Materials and Methods) and fat pad weights were not adjusted for lean body weight. Total fat pad weight is the sum of the three fat pad weights. Adiposity index was calculated by dividing total fat pad weight by body weight. The mean with stars (*, P < 0.05; **, P < 0.01; ***, P < 0.001) within the same sex is significantly different from that of B6 (Dunnett's test).

To narrow the genomic region that affects obesity, a phenotypic comparison was made among five subcongenic strains with overlapping and non-overlapping wild-derived genomic regions (Figure 1) fed standard chow. This comparison indicated that at least one locus that affects obesity is located within the region common to B6.Cg-*Pbwg1*/SR13 and B6.Cg-*Pbwg1*/SR8 but not to B6.Cg-*Pbwg1*/SR3. Since the body weight traits of B6.Cg-*Pbwg1*/SR8 and B6.Cg-*Pbwg1*/SR12 were significantly different from that of B6 (Table 2), we adjusted obesity-related traits for lean body weight. A comparison of the adjusted data among the five subcongenic strains supported the results for the above unadjusted data (data not shown).

**Figure 2:**
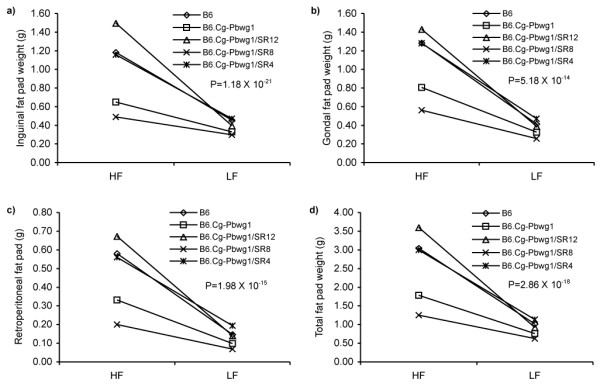
**Interaction effects of strain by diet on fat pad weights**. Inguinal (a), gonadal (b), retroperitoneal (c) and total (d) fat pad weights. LF: low-fat standard chow diet; HF: high-fat diet.

Similar to the standard chow diet, the B6.Cg-*Pbwg1*/SR8 strain fed a high-fat diet showed significantly lower body weight and obesity-related traits (Table 3). When the results obtained from standard chow and high-fat diets were combined and reanalyzed, significant interactions between diet and strain (P = 5.18 × 10^-14 ^- 1.18 × 10^-21^) were found for all fat pad traits (Figure 2). Among the strains examined, B6.Cg-*Pbwg1 *and B6.Cg-*Pbwg1*/SR8 mice showed strong resistance to diet-induced obesity compared to B6 and two positive control subcongenics, B6.Cg-*Pbwg1*/SR4 and B6.Cg-*Pbwg1*/SR12.

**Table 3:** Obesity traits in selected subcongenics and their parental strains on high-fat diet

Strain	n	Body weight (g)	Lean body weight (g)	Inguinal fat pad weight (g)	Gonadal fat pad weight (g)	Retroperitoneal fat pad weight (g)	Total fat pad weight (g)	Adiposity index
B6	15	32.318 ± 0.165	29.21 ± 0.127	1.203 ± 0.024	1.294 ± 0.039	0.604 ± 0.021	3.100 ± 0.083	0.093 ± 0.002
B6.Cg-*Pbwg1*	12	29.752 ± 0.047*	28.01 ± 0.149	0.625 ± 0.023***	0.794 ± 0.037***	0.327 ± 0.020**	1.745 ± 0.081***	0.058 ± 0.002***
B6.Cg-*Pbwg1*/SR12	11	34.028 ± 0.149	30.44 ± 0.133	1.495 ± 0.019*	1.418 ± 0.013*	0.673 ± 0.011	3.586 ± 0.044	0.105 ± 0.001
B6.Cg-*Pbwg1*/SR8	13	28.058 ± 0.186**	26.84 ± 0.147*	0.478 ± 0.034***	0.547 ± 0.043***	0.196 ± 0.025***	1.221 ± 0.098***	0.043 ± 0.003***
B6.Cg-*Pbwg1*/SR4	10	31.861 ± 0.234	28.89 ± 0.145	1.134 ± 0.033	1.274 ± 0.052	0.563 ± 0.027	2.970 ± 0.112	0.092 ± 0.003

Data are least-squared means ± SE at 13 weeks of age. n, number of mice. Data were analyzed with a linear model (see Materials and Methods) and fat pad weights were not adjusted for lean body weight. Total fat pad weight is the sum of the three fat pad weights. Adiposity index was calculated by dividing total fat pad weight by body weight. The mean with stars (*, P < 0.05; **, P < 0.01; ***, P < 0.001) within the same sex is significantly different from that of B6 (Dunnett's test).

From results of the above analyses, the obesity locus was physically defined within an approximately 8.8-Mb region between *D2Mit270 *and *D2Mit472*. This region, derived from the wild mouse, was resistant to high-fat diet and contained the positions of peak LOD scores for four previously identified fat pad QTLs, *Pbwg1.5 *- *Pbwg1.8 *(Figure 1).

### Metabolic profiling of selected subcongenics

**Figure 3:**
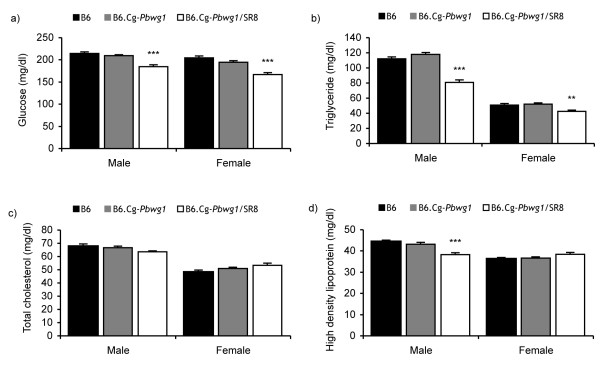
**Metabolic profiles of selected subcongenics and their background strain, B6**. Glucose (a), triglyceride (b), total cholesterol (c) and high density lipoprotein (d) in serum. Numbers of mice examined were: 9 males and 5 females for B6, 7 males and 7 females for B6.Cg-*Pbwg1*/SR4, and 8 males and 8 females for B6.Cg-*Pbwg1*/SR8 mice. Data are means ± SE. Star marks above the bars within a sex indicate a significant difference from B6 mice (**, P < 0.01; ***, P < 0.001, Dunnett's test).

Since the B6.Cg-*Pbwg1*/SR8 subcongenic strain showed significant reductions in body weight and obesity-related traits, the serum levels of glucose, triglyceride, total cholesterol and high density lipoprotein were investigated in this strain in comparison with those in B6 and B6.Cg-*Pbwg1*/SR4, a positive control, at 13 weeks of age on a standard chow diet. In both sexes, glucose and triglyceride levels were significantly lower in the B6.Cg-*Pbwg1*/SR8 strain than in the B6 strain (Figure 3a, b). No significant differences were observed in total cholesterol level between the B6 strain and each of the subcongenic strains (Figure 3c). However, the serum level of high density lipoprotein was lower in males, but not in females, of the B6.Cg-*Pbwg1*/SR8 strain than in males of the B6 strain (Figure 3d).

### Candidate gene analysis

According to UCSC Genome Browser, the 8.8-Mb region between *D2Mit270 *and *D2Mit472 *contained 29 genes. Microarray analysis revealed that four genes in the liver and seven genes in the gonadal fat pad were differentially expressed in B6.Cg**-***Pbwg1*and B6 strains at a signal log ratio ≥ 1.0 (Table 4). *Gpd2 *was upregulated in both the liver and fat of B6.Cg-*Pbwg1 *mice, and *Acrv1c *was upregulated in fat only. On the other hand, *Upp2*, *March7 *and *Rbms1 *were downregulated in the liver and/or fat of B6.Cg-*Pbwg1 *mice.

**Table 4:** Differentially expressed genes in livers and gonadal fat pads of B6.Cg-*Pbwg1 *and B6 mice

Organ	Probe name	Gene symbol	Gene name	Alignments (bp)^a^	Signal Log Ratio^b^
Liver	1452741_s_at	*Gpd2*	Glycerol phosphate dehydrogenase 2, mitochondrial	chr2: 57219081-57223134	1.5
	1424969_s_at	*Upp2*	Uridine phosphorylase 2	chr2: 58607353-58643653	-1.0
	1440966_at	*March7*	Membrane-associated ring finger (C3HC4) 7	chr2: 60084730-60086015	-2.3
	1434005_at	*Rbms1*	RNA binding motif single stranded interacting protein 1	chr2: 60588251-60590840	-1.0

Gonadal fat pad	1452741_s_at	*Gpd2*	Glycerol phosphate dehydrogenase 2, mitochondrial	chr2: 57219081-57223134	1.0
	1443225_at	*Acvr1c*	Activin receptor type 1C	chr2: 58124237-58124901	1.5
	1440966_at	*March7*	Membrane-associated ring finger (C3HC4) 7	chr2: 60084730-60086015	-1.7

^a ^Based on NCBI 37 mouse genome assembly in UCSC Genome Browser. ^b ^A positive value indicates an upregulated gene in B6.Cg-*Pbwg1 *mice, whereas a negative value indicates a downregulated gene in B6.Cg-*Pbwg1 *mice.

## Discussion

In this study, we revealed that an obesity QTL is located within an approximately 8.8-Mb region between *D2Mit270 *(52.76 Mb) and *D2Mit472 *(61.54 Mb) on mouse chromosome 2 and that the QTL effect is resistant to both standard and high-fat diets. Four closely linked obesity QTLs (*Pbwg1.5 *for gonadal fat pad weight, *Pbwg1.6 *for inguinal fat pad weight, *Pbwg1.7 *for retroperitoneal fat pad weight and *Pbwg1.8 *for total fat pad weight) that we found previously reside within this region [6]. Although it was not able to be concluded in the present study whether these four QTLs are the same or different loci, we verified all phenotypic differences defined by our previous study. We are now developing other subcongenics with small regions into which the 8.8-Mb target region will be divided. Phenotypic characterization of those subcongenics will provide a clue for resolving the issue of pleiotropy vs. closely linked loci.

Lipid profiling, especially triglyceride level, is an indirect but well-known measure of obesity, and a strong positive correlation exists between obesity and triglyceride level [18]. On the other hand, a high glucose level in plasma is an indicator of diabetes [19]. Since the genetic background of the B6 strain is susceptible to diet-induced obesity and diabetes, in order to investigate the primary biological function of the present obesity QTL, we measured glucose, triglyceride, total cholesterol and high density lipoprotein levels in serum along with direct measurement of the weight of individual fat depots. Significantly lower glucose and triglyceride levels in B6.Cg-*Pbwg1*/SR8 subcongenic mice than in the B6 mice suggest that the locus might specifically regulate the energy metabolism and storage or differentiation of adipose tissue. On the other hand, insignificant differences in total cholesterol level between B6 and B6.Cg-*Pbwg1*/SR8 mice in both sexes suggest that the cholesterol metabolism may be independent of obesity and the gene(s) related to the cholesterol metabolism might reside outside of the present obesity QTL region.

Body weight, obesity and related comorbidities are highly influenced by diet [20], and there is discordance between obesity and related phenotypes [21]. Therefore, we tested the effect of the present obesity QTL on high-fat diets. The findings of the present study agreed well with results of studies by Cheverud et al [20,21] showing significant variation between strains in response to high fat feeding.

Many independent studies with different mouse models mapped several QTLs for body weight, growth, adiposity and related traits on mouse chromosome 2 [2]. Among QTLs investigated in other studies, *Nidd5*, which controls body weight and adiposity, was physically defined to be in a 9.4-Mb interval between *D2Mit433 *(57.2 Mb) and *D2Mit91 *(66.6 Mb) by an analysis using congenic mouse strains [8]. This region partially overlaps with our *D2Mit270 *(52.76 Mb)-*D2Mit472 *(61.54 Mb) region. In addition, *Bw77 *and *Niddm46 *both affecting body fat amount in rats [22-24], and *BW374_H *for abdominal visceral fat in humans [22,25] are mapped to corresponding conserved syntenic intervals for our region. Concordance of QTLs from different genetic studies in mice and other species increases the possibility of having common and strong candidate genes within the chromosomal region of interest [26].

To date, most of the body weight, obesity and diabetes QTL mappings in the mouse have been performed in populations obtained from crosses between common inbred strains that are largely descended from *M. m. domesticus *[8,20]. Consequently, the obesity QTLs identified are restricted within a small gene pool of ancestors of the inbred strains. In this study, we used wild *M. m. castaneus *mice captured in the Philippines that have only 60% of the body weight of the B6 strain [17]. The obesity-resistant QTL allele derived from the wild *castaneus *mouse must reflect variation in nature, because males obtained from a cross of wild-caught mice were directly used to map QTLs for body weight and growth [17] and thereafter some of the wild-derived QTL alleles discovered have been saved by development of congenic strains [6,27,28]. Other studies using wild mice, including *M. m. molossinus *and *M. m. castaneus*, have revealed several loci related to growth, obesity, aging, abnormal spermatogenesis, and diabetes [29-33]. Thus, a number of novel QTLs with unique phenotypic effects may remain undiscovered from the gene pools of wild mouse populations.

The use of mice as a model animal for studying genetic influences on obesity is based on the premise that mice and humans share common regulatory systems of body weight and fatness. To date, this has proved to be the case and there are many examples of correspondence in particular genes and phenotypes in mice and humans. For example, in mice, mutations in the leptin gene cause early-onset, extreme obesity and the same is true for humans [34,35]. Targeted genetic manipulation in mice has also established vital regulatory roles of molecules in obesity such as the melanocortin 4 receptor (MC4R) [36]. Pro-opimelanocortin, which produces a ligand for MC4R α-melanocyte-stimulating hormone, is also involved in human obesity [37].

The region of mouse chromosome 2 reported here has a conserved synteny with the human chromosome 2 region, 2q23.3-q24.2 (154333852 to 162272605 bp). According to the public mouse genome database (NCBI Build 37 on UCSC Genome browser), 29 known protein-coding genes are present within the candidate interval between *D2Mit270 *and *D2Mit472*. Since 25 of these genes are also found in the human chromosomal region (2q23.3-q24.2), no homologues of *A930012016Rik*, *AL929070.15*, *Dapl1 *and *AL928581.8-1 *were found on human chromosomes, suggesting that these four genes on mouse chromosome 2 might be nonfunctional.

Since liver and fat depots are major role players in energy metabolisms and homeostasis, genes for obesity QTLs are expected to be expressed in these tissues. Therefore, to search for specific *cis*-acting genes that could account for the present results, we performed microarray analysis using the B6.Cg-*Pbwg1 *congenic strain and its background B6 strain. This analysis can minimize the *trans*-effects of genes located outside of the congenic region because the congenic and B6 strains have generally the same DNA sequences except for that region. However, *trans*-acting genes within the region and environmental factors may affect the gene expression levels obtained. Furthermore, we cannot completely rule out the possibility that additional genes on the unwanted donor regions that could not be eliminated by recurrent backcrossing during development of the congenic strain might still affect the gene expression levels. Thus, we are now planning to perform a new gene expression study using an F2 segregating population between a developed subcongenic strain and the B6 strain. The use of the F2 can cancel out the possible effects of *trans*-acting genes on the unwanted donor regions because their alleles are segregating regardless of diplotype for the subcongenic region of the F2.

**Figure 4:**
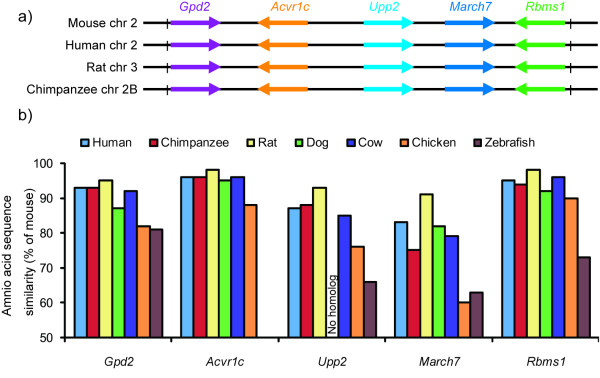
**Evolutionary conservation of candidate genes**. Gene order, transcriptional direction and chromosomal localization of the candidate genes in different mammalian species (a). The positions of the transcripts were arbitrarily drawn and the arrowhead indicates transcriptional direction. The % similarity of amino acid sequences of the candidate genes between the mouse and other organisms (b) (HomoloGene Release 64, http://www.ncbi.nlm.nih.gov/homologene/.

Our microarray analysis revealed five genes, *Gpd2*, *Acvr1c*, *Upp2*, *March7 *and *Rbms1*. These genes are highly conserved among different species in terms of gene order, relative transcription direction, sequence similarity, and functional relationships as inferred by gene ontology (Figure 4). Several reports suggest that the sequence of a functionally more important gene has a low rate of evolution such that its orthologs from distantly related species are detectable and alignable [38-40]. Comparative sequence analysis of these genes indicated that they are highly conserved and found from human to zebrafish (Figure 4b). Therefore, we can postulate that these five genes might be involved in obesity. The gene *Gpd2 *is involved in gluconeogenesis, energy production, and thermogenesis [41,42]. *Acvr1c *is a type I receptor for the TGFB family of signalling molecules. Upon ligand binding, type I receptors phosphorylate cytoplasmic SMAD transcription factors, which then translocate to the nucleus and interact directly with DNA or in complex with other transcription factors [43]. The functions of *Upp2*, *March7 *and *Rbms1 *are not well understood now.

We also compared the available genomic DNA sequences of the CAST/Ei strain derived from wild *M. m. castaneus *mice trapped in Thailand and the B6 strain using a database [44]. As 286 mutations were discovered in both coding and UTR regions of genes within our target chromosomal region, it was very difficult to make a succinct conclusion about the candidacy of our QTL on the basis of such sequence differences only. Since the DNA sequence of our *castaneus *mouse is unknown and the origin and breeding strategies are quite different from those of CAST/Ei, we are now planning to sequence genomic DNA and cDNA sequences of the five differentially expressed genes mentioned above to find any mutations, substitutions or deletions in the genes.

## Conclusions

By using a series of subcongenic strains, we physically delineated the region of an obesity QTL previously discovered from an untapped resource of the wild *M. m. castaneus *mouse to an approximately 8.8-Mb interval between *D2Mit270 *and *D2Mit472 *on mouse chromosome 2. The wild-derived QTL allele prevents obesity in mice fed both standard and high-fat diets. A further understanding of this unique QTL effect at genetic and molecular levels may lead to the discovery of new biological and pathologic pathways associated with obesity.

## Methods

### Construction of subcongenic strains

B6.Cg-*Pbwg1 *mice [6,17] were crossed to B6 mice purchased from Clea Japan (Tokyo, Japan), and F1 mice obtained were intercrossed to generate F2 mice. The F2 mice were genotyped for 25 microsatellite markers (Table 1) according to the method described previously [6]. Recombinant individuals were used as founders for development of subcongenic strains.

### Diet and husbandry

All pups were weaned at 3 weeks of age, and then littermates of the same sex were housed in groups of up to three mice per cage. Standard chow (CA-1, Clea Japan, Tokyo, Japan), containing 27.3% crude protein, 5.1% crude fat, 3.7% crude fiber, 7.6% crude ash and 3.45 Kcal/g energy, and tap water were provided *ad libitum*. To assess the response to high-fat feeding, a group of male mice from the B6.Cg-*Pbwg1 *strain and three subcongenic strains, B6.Cg-*Pbwg1*/SR4, B6.Cg-*Pbwg1*/SR8 and B6.Cg-*Pbwg1*/SR12, were fed a purified high-fat diet with 45% energy from fat (24% crude fat and 4.73 Kcal/g energy) (Research Diets D12451, Research Diets, New Brunswick, NJ, USA) for 7 weeks from 6 weeks to 13 weeks of age. All mice were reared in an environment with a temperature of 23 ± 3°C, 55% relative humidity, and a light/dark cycle of 12:12. This study was carried out according to the guidelines for the care and use of laboratory animals of the Graduate School of Bioagricultural Sciences, Nagoya University, Japan.

### Phenotypic characterization

Although our previous QTL analysis for obesity-related traits was performed at 10 weeks of age [6], in this study we characterized 273 mice in all subcongenic strains and 182 mice in their parental strains, B6.Cg-*Pbwg1 *and B6, at 13 weeks of age because phenotypic expression of obesity-related traits was more prominent in older mice. To avoid an arbitrary effect of feed intake on phenotypic values, mice were fasted for four hours and were anesthetized by ether anesthesia before measuring traits as described below. After recording body weight, blood was collected immediately by retro-orbital puncture. Total body length (from the tip of the nose to the end of the tail) and tail length (from the anus to the end of the tail) were measured. Head-body length was obtained by subtracting tail length from total body length. Right and left sides of inguinal, gonadal (epididymal in males and parametrial in females), and retroperitoneal white fat pads were dissected and weighed. The sum of the above three fat pad weights was considered as total fat weight in this study, and the adiposity index was calculated by dividing total fat pad weight by body weight. Lean body weight was computed by subtracting total fat pad weight from body weight. The same person recorded all phenotypic data in order to minimize variation in dissecting techniques. For metabolic profiling of animals, the serum levels of glucose, triglyceride, total cholesterol and high density lipoprotein were determined with the plate reader Sunrise-Basic TECAN (Tecan Japan, Kawasaki, Japan) using glucose CII-test, triglyceride E-test, total cholesterol E-test and HDL-C test Wakos (Wako pure chemical, Osaka, Japan), respectively.

### Microarray analysis

Livers and gonadal fat pads of three males at 10 weeks of age in each of the B6.Cg-*Pbwg1 *and B6 strains were collected and immediately immersed in RNAlater reagent (Ambion, Tokyo, Japan). Equal amounts of the three individual samples collected were pooled by tissue and strain. Total RNA extraction with TRIzol reagent (Invitrogen, Tokyo, Japan) and microarray analysis with the Affimetrix GeneChip^® ^Mouse Genome 430 2.0 array were commissioned to the Bio-medical Department of Kurabo Industries, Ltd. (Osaka, Japan)

### Statistical analyses

Sex-separated phenotypic data from the standard chow diet were analyzed by using a linear mixed model of Lme4 package implemented in R http://www.r-project.org[45]. Effects of strain, parity, litter size and number of animals per cage were treated as fixed effects and the effect of dam was considered as a random effect, and finally the best suitable model was selected on the basis of AIC (Akaike's information criterion) [46]. On the other hand, data from the high-fat diet were analyzed with a linear model that included the effects of strain, parity and litter size as fixed effects. The covariates and their interactions that had significant effects at the nominal 5% level were included in the final model for comparisons of phenotypic data from the high-fat diet among subcongenics and B6. Metabolic profile data were analyzed by one-way analysis of variance. Differences of B6 vs. congenic or subcongenic strains were determined using Dunnett's *post-hoc *test.

## Authors' contributions

MBRM carried out the genotyping, phenotyping, statistical analyses and drafting of the manuscript. AI conceived and supervised the study, participated in the design and coordination, created the subcongenic strains and helped to draft the manuscript. All authors read and approved the final manuscript.
